# isomiRs–Hidden Soldiers in the miRNA Regulatory Army, and How to Find Them?

**DOI:** 10.3390/biom11010041

**Published:** 2020-12-30

**Authors:** Ilias Glogovitis, Galina Yahubyan, Thomas Würdinger, Danijela Koppers-Lalic, Vesselin Baev

**Affiliations:** 1 Faculty of Biology, University of Plovdiv, Tzar Assen 24, 4000 Plovdiv, Bulgaria; ilias@uni-plovdiv.bg (I.G.); gyahubyan@uni-plovdiv.bg (G.Y.); 2 Department of Neurosurgery, Cancer Center Amsterdam, Amsterdam University Medical Centers, VU University Medical Center, De Boelelaan 1117, 1081 HV Amsterdam, The Netherlands; t.wurdinger@amsterdamumc.nl (T.W.); d.lalic@amsterdamumc.nl (D.K.-L.)

**Keywords:** isomiRs, microRNAs, NGS, tools, data analysis

## Abstract

Numerous studies on microRNAs (miRNA) in cancer and other diseases have been accompanied by diverse computational approaches and experimental methods to predict and validate miRNA biological and clinical significance as easily accessible disease biomarkers. In recent years, the application of the next-generation deep sequencing for the analysis and discovery of novel RNA biomarkers has clearly shown an expanding repertoire of diverse sequence variants of mature miRNAs, or isomiRs, resulting from alternative post-transcriptional processing events, and affected by (patho)physiological changes, population origin, individual’s gender, and age. Here, we provide an in-depth overview of currently available bioinformatics approaches for the detection and visualization of both mature miRNA and cognate isomiR sequences. An attempt has been made to present in a systematic way the advantages and downsides of in silico approaches in terms of their sensitivity and accuracy performance, as well as used methods, workflows, and processing steps, and end output dataset overlapping issues. The focus is given to the challenges and pitfalls of isomiR expression analysis. Specifically, we address the availability of tools enabling research without extensive bioinformatics background to explore this fascinating corner of the small RNAome universe that may facilitate the discovery of new and more reliable disease biomarkers.

## 1. Multi-Layered *miR* Gene Control as a Source of Plentiful miRNA Sequence Variations

MicroRNAs (miRNAs) were discovered in the soil-dwelling nematode *Caenorhabditis elegans* to mediate its temporal pattern formation [1,2]. Since then, thousands of miRNAs have been identified and characterized in animals, plants, and some animal viruses [3]. The latest release of the miRNA repository miRBase (v22) contains 1917 human hairpin precursors and 2654 mature sequences [4]. MiRNAs are thought to collectively regulate one-third of human genes, thereby acting as key regulators of development, cell differentiation, and homeostasis [5].

Most miRNAs are typically 21–24 nt in length and encoded by their own genes (*MIR* genes). The encoding loci of some miRNAs reside in introns or untranslated regions of protein-coding genes, as well as in introns or exons of non-coding RNAs [6,7]. Certain miRNA genes have been found clustered and being transcribed as monocistronic or polycistronic transcripts [8].

### 1.1. Canonical miRNA Biogenesis Pathway

*MIR* genes are transcribed in a manner similar to protein-coding genes by RNA polymerase-II (Pol II), and occasionally by RNA polymerase III (Pol III) to yield primary miRNAs (pri-miRNAs) and undergo multistep processing [9,10].

Each pri-miRNA forms a hairpin-shaped structure which is processed further by a microprocessor complex, consisting of one molecule of the ribonuclease III (RNase III) enzyme Drosha and two molecules of DGCR8 (Di George Syndrome critical region gene 8) [11]. DGCR8 directs Drosha to the double stranded RNA (dsRNA)–single stranded RNA (ssRNA) junction on the pri-miRNAs to cleave 11 bp away from the junction releasing ∼50–80 nt long precursor miRNAs (pre-miRNAs). The newly produced pre-miRNA is transported to the cytoplasm via exportin-5 [10] and RAN–GTP [12,13,14].

The RNase III enzyme Dicer, together with the dsRNA-binding proteins TRBP (transactivation response element RNA-binding protein) and/or PACT (protein activator of the interferon-induced protein kinase), then further cleaves pre-miRNA to generate a short double-stranded RNA fragment [15,16,17]. The miRNA duplex has a ∼2 nt 3′overhangs produced by the consecutive cleavage of Drosha and Dicer [18,19]. Once formed, the duplex is loaded into an Argonaute (AGO) protein where one of the duplex strands is retained (guide strand, miR) and the other is expelled and degraded (passenger strand, miR*) [20]. Usually, the retained strand is the one that has the less stably base-paired 5′ end in the duplex [21,22] and usually with a 5′-terminal pU or pA [23,24].

In humans, the AGO family comprises four family members (Ago1–4), of which the Ago2 is the most abundant [25]. AGO and the retained ssRNA form the mature miRNA-induced silencing complex (miRISC). Recently, it was reported that the half-lives of miR and miR* strands for some miRNA duplexes were similar, indicating similar loading onto miRISC [26].

### 1.2. Non-Canonical miRNA Biogenesis Pathways

Dicer-independent pathway: Like the canonical pathway, the first cleavage step of miRNA processing is carried out by Drosha and results in hairpin-shaped pre-miRNA. There are examples such as that of miR-451, in which Drosha cleavage yields a short pre-miRNA (42 nt) with only a 17 nt stem that is of insufficient length to be a Dicer substrate [27]. Unlike the canonical pathway, the entire pre-miRNA instead of binding to Dicer is loaded on AGO2, which is the only one of all AGO proteins that has a slicer activity, and the second cleavage step of the 3p strand is performed followed by 3′-5′ trimming to complete the miR-451 maturation [28,29].

Drosha-independent pathway: The best characterized Drosha-independent miR biogenesis pathway is that of mirtrons-introns with hairpin potential [30,31,32]. Unlike the canonical pathway, pre-miRNA-like hairpins are generated by the splicing machinery and lariat-debranching enzyme, without the need for Drosha’s involvement [30,31].

### 1.3. IsomiR Biogenesis Pathways

**Figure 1:**
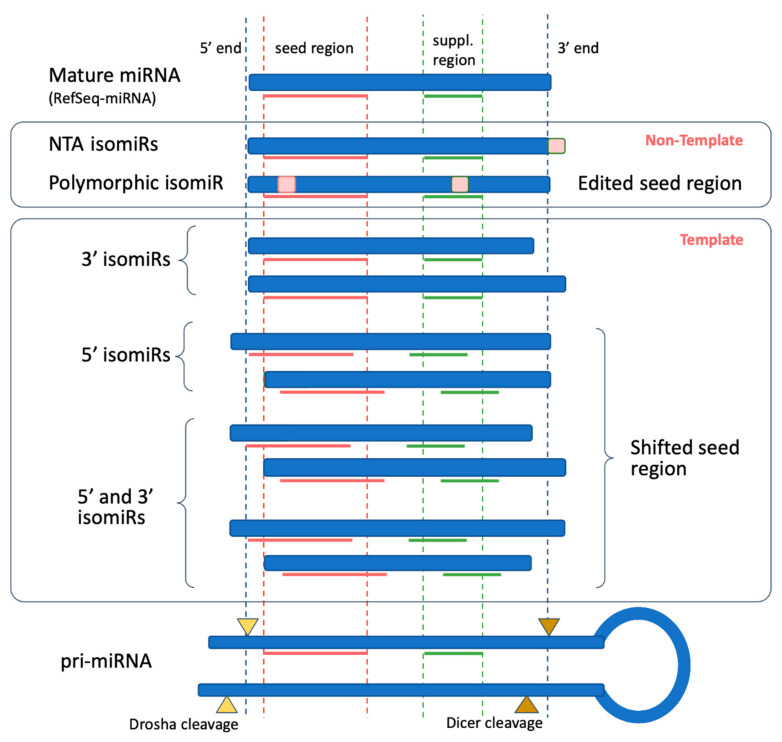
Length and sequence variations of isomiRs associated with the mature microRNA (miRNA) sequence (reference sequence, RefSeq) in miRBase.

It has long been thought that each arm of the pre-miRNA produces one miRNA (miR and miR*). High-throughput sequencing has revealed a variety of mature miRNA forms called isomiRs that are produced from a single pre-miRNA and differ in length, sequence or both [33,34,35,36,37]. Small RNA databases annotate one “mature sequence” per pre-miRNA arm, which is usually the most frequent sequence across all deposited sequences at the time of entry into the database [38]. Appropriately, it has been suggested by Desvignes and colleagues (2015) to designate this molecule as “reference sequence miRNA” (RefSeq-miRNA) to which any newly identified variant is assigned. The number of microarray and sequencing assays of healthy and pathological human tissue have revealed specific isomiRs that are substantially more abundant than the RefSeq-miRNA (Figure 1) [39,40].

Depending on the degree of matching with pri-miRNA, isomiRs are classified as template (if there is a complete match with pri-miRNA) or non-template (if the match with pri-miRNA is not complete isomiRs. Regarding the template isomiRs, comparing the locations of the RefSeq and the isomiR sequence on the pri-miRNA shows whether the 5′-, 3′-, or both ends are shifted simultaneously. 5′-isomiRs, and some of the polymorphic isomiRs, have an altered seed region, and so may target different mRNA molecules.

Alternative cleavage by Drosha and/or Dicer generates templated isomiRs with variable 5′ and/or 3′ends: IsomiRs production is attributed mainly to imprecise or modulated variations in cleavage by Drosha and/or Dicer during miRNA biogenesis [35]. The selection of cleavage sites by the two RNase III enzymes defines the miRNA 5′- and 3′-ends. Drosha and Dicer recognize specific pri-RNA and pre-RNA structural modules and sequence motifs that set the cleavage distance [41,42]. Some pri-miRNAs are processed by Drosha at multiple sites, producing more than one pre-miRNA and mature miRNA species from a single primary transcript [43,44]. Dicer’s activity can be modulated by its partner proteins TRBP and PACT. Competing for Dicer binding, under different conditions or in different cell types, TRBP and PACT can control dsRNA recruitment, relative orientation, and conformational changes, and thus isomiR production [17].

Non-templated nucleotide additions (NTA) produce 3′-tailed isomiRs: NTA mainly involves adenylation or uridylation at miRNA 3′-ends by multiple terminal nucleotidyl transferases [45]. Mono- and oligo-adenylation is carried out by the poly(A) RNA polymerase (PAP) associated domain-containing proteins PAPD4 (also known as GLD2) and PAPD5 [46,47,48,49]. Uridylation of miRNAs and their sequence variants can be mediated by terminal uridylyl transferases (TUTases) including TUT4 and TUT7 (also known as Zcchc11 and Zcchc6, respectively) in humans [50,51,52,53]. In hsa-let-7 biogenesis, the switch from mono- to oligo-uridylation of pre-*let*-7 is controlled by the RNA-binding protein Lin28 and represses the expression of mature let-7 [50,51].

A-to-I modification of the sequence of miRNA precursor creates edited miRNA sequence variants: Other miRNA isoforms are also generated from adenosine-to-inosine (A-to-I) RNA-editing by adenosine deaminase acting on RNA-ADARs (ADAR1 and ADAR2 in mammals) which can recognize the double-stranded structure of pri-miRNAs [54]. A-to-I modifications of pri-miRNA can interfere with the pri-miRNA and/or pre-miRNA processing [54] and lead to miRNA sequence variations [55]. Since inosine I is recognized as guanosine (G) [56], editing of the miRNA seed (at positions 2–7) can redirect the miRNA to a new set of targets [55].

### 1.4. Mode of miRNA Action (miRNA Mediated Gene Expression Regulation)

MiRISC is the effector complex of miRNA-mediated gene expression regulation, where miRNAs function as a specificity determinant while AGO exerts the regulatory effect [57]. MiRNAs have a predominantly inhibitory effect on the expression of their effector genes, and rarely positive, acting at both the post-transcriptional and transcriptional levels.

Most studies have shown that canonically, miRNAs guide miRISC to a specific target mRNA through sequence-specific base pairing with complementary sites present in the 3′-untranslated region (UTR) [58]. AGO recruits a member of the glycine-tryptophan (GW) protein family (TNRC6A/GW182, TNRC6B, and TNRC6C in humans) [59], which in turn interacts with other effector proteins to cause mRNA decay or translation repression [60,61,62].

Some studies have revealed activating regulation of gene expression by miRNAs. During the miRNA-mediated enhancement of translation, AGO2 interacts with FXR1 (fragile X mental retardation protein 1) instead of GW182 as it was shown in quiescent cells and oocytes [63,64]. Activating miRNA was found to bind to 3′UTR [65] as well as to 5′UTR [66].

The number of studies reporting nucleus-enriched miRNA with functional significance is increasing. In the nucleus, miRNAs are thought to act co-/post-transcriptionally regulating target mRNA stability of and splicing events or may regulate transcription directly by inducing epigenetic alterations at specific gene promoters.

### 1.5. IsomiR Biological Implication

IsomiR variations can differ between tissue types, disease states, and change across developmental time [39,67]. 3′ isomiRs, the most abundant miRNA sequence variants, can exhibit mainly altered stability [46,68], and sub-cellular localization [69,70], and are less likely to affect target selection [71]. Both 5′ isomiRs and A-to-I edited variants can have altered seed region and so may target the same or different mRNA molecules and biological pathways compared to the corresponding RefSeq-miRNA [55,72,73,74].

## 2. Bioinformatics Approaches of Identifying and Analyzing miRNA and isomiR Molecules

Beyond quantitative evaluation, next-generation sequencing (NGS) allows for single-base resolution of known and novel miRNAs, and it is currently used to identify context-specific miRNA species in samples. For analyzing small RNA (sRNA) NGS data, a variety of bioinformatics tools have been developed allowing profiling the miRNA repertoire, discovering new potential miRNAs as well as identification of their isoforms. In the beginning of miRNA profiling era, the detected 5′- and 3′-sequence variations were treated as artifacts and discarded from the downstream analysis [75,76]. However, miRNA sequence variation phenomenon was quickly acknowledged as part of the comprehensive miRNA biogenesis landscape [45,77,78]. Thus these length and sequence variants were named isomiRs, and gradually became the main objectives for the miRNA analysis software [33]. For the past decade, many tools have been specifically tailored to miRNA analysis but exploring them in-depth shows quite different performances in terms of sensitivity and accuracy levels as well as used methods, workflows, and processing steps, and at the end output datasets have variable overlapping. In this regard, many issues remain bioinformatics challenges, such as (i) the nature of sRNAs being that they are a quite small molecule and mapping procedure can lead to mapped sequence at the wrong location, and (ii) some *MIR* genes having duplication which can lead to difficulties on the analysis steps.

Almost all analyzing tools of miRNA NGS data are performed using Linux/Unix servers or clusters. However, analyzing and outputting results in Linux is difficult for most biology scientists. Furthermore, the process steps of the analysis include command line workflows and produce large amounts of data and files that are difficult to perform and interpret. It is therefore quite important to develop efficient tools that have a user-friendly GUI (graphic user interface) and good visualizations that provide better human-readable summarization of the results. On the other hand, there are two major problems with implementing such tools: (i) web-based tools have some restrictions, such as limited data upload, providing data on a lab-external computer, and the tool access and support depend largely on the author, and (ii) stand-alone programs may require extra steps by the user to run and fulfill the requirements, and may also be quite overwhelming in terms of computer power.

There are also challenges regarding the specificity of the detection of isomiRs due to their heterogeneous origin. Several combinations of 5′ and 3′ trimmed and tailed, templated and non-templated, isomiRs can exist at once, making the overall analysis more complex. Development of a single algorithm capable of processing all detectable miRNA variants as isomiRs can be nearly impossible [79]. Several in silico software tools already support isomiR identification at different levels. Nevertheless, among them, there is a lack of consensus on isomiR classification and nomenclature. Furthermore, some tools are only able to partially identify the repertoire of isomiRs or perform only isomiR discovery without the capability of extracting differentially expressed isoforms.

**Figure 2:**
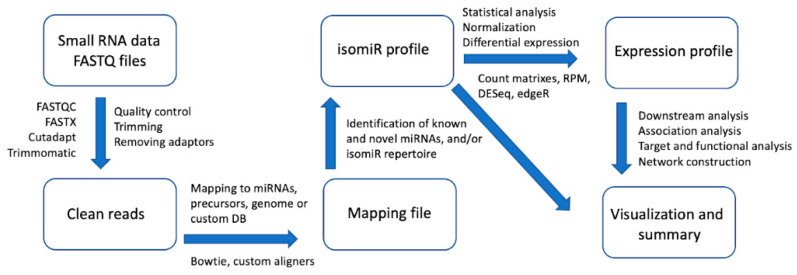
General summary of the workflow used in miRNA and isomiR analysis tools.

As there is no consensus on the steps for analyzing miRNAs and their isoforms, we present here a summary diagram of the major processing steps used in most tools for identification and/or analysis of isomiR in sRNA-seq datasets (Figure 2). Most of the tools (CBS-miRSeq, iMir, SRNAnalyser, MIRPIPE, MiRge, QuickMIRSeq, MirDis, Jasmine, miRMaster, OptimiR, and Prost!), always include a pre-processing step in which the raw reads are quality checked, trimmed, and cleaned, allowing reads to be used in the next steps. Widely accepted external tools for this step are, for example, FastQC, Cutadapt, and Trimommatic. In the next step, these clean sRNA reads have to be mapped on the reference database. For the specifics of isomiR identification, and the study of the sequence variant set that a particular miRNA has, the reference database can be the pre-miRNA database (usually using miRBase and miRGene), reference genome, or custom-made database. For this step, a wide range of tools are developed on top of the widely used Bowtie aligner. Few tools, such as miRPro and Chimira, use Novoalign and BLAST as mappers and specifically created custom aligners (CASMIR, isomiR-SEA). Firstly, the reference sequence of the mature miRNA is localized on the pre-miRNA and then all other molecules that map to a defined region comprising miRNA site can be explored to detect all types of 3′ and 5′ offset and modified sRNAs thus allowing a precise isomiR classification. Some tools use a different approach by creating a custom database with miRNA seed/motif sequences that are subsequently used for the mapping (for example, isomiR-SEA, QuagmiR). It is important to note that since there is a lack of consensus on isomiR nomenclature and classification, some tools do not provide a detailed and comprehensive analysis of individual isomiRs but rather explore the patterns in the whole subset of molecules. For this reason, only a few tools provide a comprehensive analysis of specific differentially expressed isomiRs in multiple samples (Table 1). When it comes to differential expression analysis, the majority of the developed software tools use external dedicated statistical packages like DESeq2 and EdgeR (iMir, miR-isomiRExp, CSB-miRSeq, sRNAtoolbox, miRpro, miRDis, Chimira, etc), or RPM normalization (DeAnniso, IsomiRage). Overall, a few tools provide distinctive features such as comprehensive isomiR profile exploration (for example CASMIR, miRMaster, IsomiR-SEA, Chimera), isomiR nomenclature (Jasmine), or even differential expression profiles and analysis at the level of a single isomiR across samples (DeAnniso, MIR-isomiRexp). Some programs additionally provide other well implemented distinctive features such as miRNA arm switching detection, isomiR analysis independent of miRNA locus, target alteration analysis, etc. (Table 1).

Due to the variety of the output files of commonly used tools, comparisons between the results of different tools are not accurate. The miRNA Transcriptomic Open Project (miRTOP) overcomes the above problem by generating a new file format miRGFF3 from the most state-of-the-art tools output files (e.g., sRNAbench, Prost!, and OptimiR). It can also convert miRGFF3 into other formats as FASTA and VCF, allowing users to continue with various analyses without being limited to only one tool.

In terms of implementation, most of the tools are web-based, providing a user-friendly interface, but limiting data size and future support, including access, are strictly dependent on the developing team. Others offer standalone tools for different operating systems (OS), but this option assumes that the user must install the tool him/herself and meet the requirements and dependencies to operate. Very few tools, for example, provide Docker images that can discard some of the mentioned disadvantages—they can provide a ready-to-run, pre-configured standalone or web-based tool that can run on a user’s computer or server, regardless of OS.

## 3. IsomiRs—A Limitless Source of Potentially Novel Biomarkers

Apart from the well-documented association of mature (e.g., RefSeq) miRNAs with diverse human diseases, recent evidence also supports dynamic isomiR repertoire in a variety of normal and diseased (pathological) tissues. Specific isomiR expression patterns have been attributed to different normal tissue types and found dependent on the tissue state, population origin, individual’s gender, and race [36,39,40,106].

IsomiRs have been associated with several human diseases such as pre-eclampsia [106], Huntington disease [107], Alzheimer’s disease [108], osteoarthritis [109], cardiovascular diseases [110], rheumatoid arthritis [111], and found to respond to bacterial infection [112]. Specific isomiR expression patterns have been observed in various cancer types [113,114] suggesting the miRNA-mediated level of regulation of tumorigenesis. In gastric cancer, several mature miRNAs generated from the two different arms (5p or 3p) of the same pre-miRNA exhibit reversed expression preferences (normal vs. cancer tissue) [115]. Next to the differential “arm-switch” expression, the enrichment of specific isomiRs produced from the same precursor may also exhibit differential expression preferences [115]. IsomiRs can function in a synergistic network with related miRNAs to suppress tumor development and progression, as it was demonstrated for miR-140-3p and its isoform in breast cancer [73], miR-451a and its isomiRs in melanoma [116], and miR-139-5p isoforms in hepatocellular carcinoma [117].

The accumulated evidence shows that the analysis of isomiRs expression pattern can differentiate tumor cells from normal, and moreover to discriminate amongst different tumor types and subtypes [118,119]. This strongly suggests isomiRs as potentially valuable diagnostic and prognostic biomarkers in cancer diagnostics [119,120,121]. Furthermore, the combination of isomiR features with state-of-the-art algorithms effectively differentiated normal from cancer cell lines [118,122]. Mature miRNAs and their isomiR variants, along with their target pathways, should be considered during tumor diagnosis, progression and treatment monitoring [114].

Several studies on body fluids such as blood (plasma and serum), urine, sputum, cerebrospinal fluid, and milk have shown an abundance not only of numerous miRNAs but also of their isomiR variants [123]. Although many of them can be found in circulation [124], enrichment of miRNAs and their isomiRs can be found in blood analytes such as human platelets (e.g., cytoplasm fragments derived from bone marrow megakaryocytes) and extracellular vesicles (EVs, e.g., secreted membrane-enclosed particles that are released naturally from the cell) [70,120,123,125]. In addition, EVs isolated from the urine of prostate cancer patients showed enrichment in miRNAs and isomiRs with potential for biomarkers in minimally invasive diagnosis of prostate cancer patients [126]. The most common feature among biofluid studies is that isomiRs have a higher abundance than known miRNAs [39,127,128]. This suggests miRNA precursors may generate more than one miRNA variants with specific properties. New in-depth studies on miRNAs and isomiRs, including the exploitation of their biosources and intricate molecular properties, in combination with the state-of-the-art computational algorithms, will aid further exploration of this rich and versatile source of potentially new biomarkers.

## Figures and Tables

**Table 1:** List of bioinformatics tools for isomiR identification and analysis in next-generation sequencing (NGS) datasets (The table can be accessed also at https://github.com/Glogobyte/isomiR_tools).

Tool Name	Type	Tool Features	Year	Authors	Journal/URL	Reference (DOI Number)
miRMaster	Web	Quantification of miRNAs and non-coding RNAs. Discovering of new miRNAs and isomiRs. Quantification on known and novel miRNA with miRDeep2. Pre-processing (adapter trimming, quality filtering, read collapsing). Mapping with Bowtie to various ncRNA (rRNAs, snRNAs, snoRNAs, scaRNAs, lincRNAs, piRNAs, tRNAs). Exogenous miRNAs mapping with bacteria and viruses. Used for validation of used samples from lung and blood samples.	2017	Tobias Fehlmann et al.	Nucleic Acids Research Open Access https://www.ncbi.nlm.nih.gov/pmc/articles/PMC5587802/	10.1093/nar/gkx595 [80]
QuickMIRSeq	Linux	Quantify miRNAs and isomiRs. Avoidance of multiple mapping issue of reads of identical sequences. Clustering and grouping of identical and similar sequences. Trimming adapters, collapsing, joint mapping with Bowtie. Remapping reads with mismatches to a reference genome to further reduce the number of false hits.	2017	Shanrong Zhao et al.	BMC Bioinformatics Open Access https://www.ncbi.nlm.nih.gov/pmc/articles/PMC5359966/	10.1186/s12859-017-1601-4 [81]
iMir	Linux Mac	Identification of miRNAs and other sncRNAs, such as piRNAs. Adapter trimming, quality filtering, differential expression analysis. Analysis of sncRNAs and novel miRNAs. Identification of isomiRs using miRanalyzer. Using miRAnalyzer and miRDeep2. Statistical analysis to remove low expressed sncRNAs. Differential expression using DESeq Quantile normalization. Target prediction using TargetScan and miRanda.	2013	Giorgio Giurato et al.	BMC Bioinformatics Open access https://bmcbioinformatics.biomedcentral.com/articles/10.1186/1471-2105-14-362	10.1186/1471-2105-14-362 [82]
MIR-isomiRExp	Web	Analyze the expression patterns or miRNA/isomer levels. MiRNA maturation and processing mechanism at isomiRs levels. Using MirBase DB, mapping with Bowtie. Differential expression using DESeq. Analysis at the isomiR levels based/independent on miRNA locus. Arm-switching analysis.	2016	Li Guo et al.	Scientific Reports Open Access https://www.ncbi.nlm.nih.gov/pmc/articles/PMC4806314/	10.1038/srep23700 [83]
miRNAgFree	Linux Windows	MiRNA prediction based on biogenesis features (known 5′ homogeneity) and isomiR duplex forming. Uses the sRNAbench preprocessing.	2017	EL Aparicio et al.	BioRxiv https://www.biorxiv.org/content/10.1101/193094v1.full	10.1101/193094 [84]
isomiRID	Linux Mac	Identification of 5′, 3′ and polymorphic isomiRs. Identification of non-templated 5′ or 3′ end and variations. It could be applied to every organism (plants and animals). Detection of isomiRs with one mismatch. Mapping in pre-miRNAs with Bowtie.	2013	Luiz Felipe Valter de Oliveira et al.	Bioinformatics https://academic.oup.com/bioinformatics/article/29/20/2521/276800	10.1093/bioinformatics/btt424 [85]
MIRPIPE	Web	Rapid and simple browser-based miRNA homology detection and quantification of miRNAs. Read counts from isomiRs of the same miRNA are combined. Using FASTX-tollkit, Cutadapt, BLASTN.	2014	Carsten Kuenne et al.	Bioinformatics https://www.ncbi.nlm.nih.gov/pmc/articles/PMC4816158/	10.1093/bioinformatics/btu573 [86]
CASMIR	Standalone, Linux	Sequence-oriented isomiR annotation which allows unbiased identification of global isomiRs from small sequencing data. Alignment against canonical and precursors from miRBase. Discovering of canonical, 5′, 3′, polymorphic, mixed type, non-templated isomiRs, quantification using miRDeep2. In-house trimming and size filtering. BLAST with in-house custom isomiR-BLAST alignment tool. Differential expression performed with a Poisson regression model combined with a quasi-likelihood approach and AUC based on methods of DeLong and Clarke-Pearson.	2018	Chung Wah Wu et al.	BMC Genomics Open Access https://www.ncbi.nlm.nih.gov/pubmed/29801434	10.1186/s12864-018-4794-7 [87]
IsomiR-SEA	Linux Windows Mac	Provide users with a complete and accurate picture of the miRNAs, isomiRs and conserved miRNA:mRNA interaction sites. Provide accurate miRNA and isomiRs expression levels. Use a specialized algorithm for alignment. Evaluates the positions of the encountered mismatches in analyzed tags.	2016	Gianvito Urgese et al.	BMC Bioinformatics https://bmcbioinformatics.biomedcentral.com/articles/10.1186/s12859-016-0958-0	10.1186/s12859-016-0958-0 [88]
DeAnnIso	Web	Detection and annotation of IsomiRs from sRNA sequencing data. Extract the differentially expressing isomiRs with isomiRs expression, isomiRs classification from paired or multiple samples. Tissue specific isomiR expression. Using Bowtie and BLAST with miRBase. Normalization with RPM. isomiRs’ classification, 5′ isomiRs, 3 isomiRs, isomiRs with internal modifications, templated and non-templated. Target analysis and enrichment analysis of isomiRs with miRanda or RNA hybrid.	2016	Yuanwei Zhang et al.	Nucleic Acids Research https://www.ncbi.nlm.nih.gov/pubmed/27179030	10.1093/nar/gkw427 [89]
IsomiRage	Windows Mac	Identification of miRNA variants (canonical miRNAs, templated and non-templated isomiRs). Detects and groups all 3′-,5′- and trimmed variants. Uses reference sequences from miRBase. Classification of isomiRs according to the type of modification (uridylation, adenylation, etc.). Mapping performed with Bowtie with no mismatches to reference and custom genome. Normalization with RPM and read counts that can be used for comparisons of fold changes and other downstream analyses.	2014	Muller Heiko et al.	Frontiers in Bioengineering and Biotechnology https://www.frontiersin.org/articles/10.3389/fbioe.2014.00038/full	10.3389/fbioe.2014.00038 [90]
QuagmiR	Standalone, Web	Designed for the detection and annotation of heterogeneous isomiRs. Provide extensive customization of the process and reference databases according to user’s diverse research needs. Can be used to analyze both private datasets and the public datasets that are available to authorized researchers through the CGC.	2018	Xavier Bofill-De Ros et al.	Bioimformatics https://www.ncbi.nlm.nih.gov/pmc/articles/PMC6499244/	10.1093/bioinformatics/bty843 [91]
Jasmine	R + Java	Identification of isomiR populations. Propose a comprehensive isomiR nomenclature. Uses curated miRBase annotation. Adapter trimming (trimmomatic, cutadapt). Collapsing trimmed reads (fastx_collapser, fastq _to_fasta from FASTX-toolkit), quality control (FastQC). Mapping with Bowtie with at least one mismatch. Uses miRBase database.	2019	Xiangfu Zhong et al.	Bioinformatics https://academic.oup.com/bioinformatics/advance-article/doi/10.1093/bioinformatics/btz806/5612093	10.1093/bioinformatics/btz806 [92]
CBS-miRSeq	Linux, Docker VM	Color-spaced raw reads. Read length distribution, summary of adapter removal, mapping statistics, and raw expression count matrix of known miRNAs. Using Bowtie with reference genome. Differential expression analysis using DESeq2, EdgeR. Identifies isomiRs using miRspring. Predicts novel miRNA candidates using miRDeep2. Predicts the most consistent miRNA:mRNA unique pairs, using RNAhybrid, miRanda.	2019	Rupesh K. Kesharwani et al.	Computers in Biology and Medicine https://www.sciencedirect.com/science/article/pii/S001048251930188X	10.1016/j.compbiomed.2019.05.019. [93]
sRNAtoolbox	Web	Expression profiling of small RNAs, prediction of novel microRNAs, analysis of isomiRs. Differential expression using DESeq, edgeR, and NOISeq. Target prediction on user-provided input data based on Miranda, PITA and TargetSpy. Visualization of sRNA expression data in a genome context using jBrowse.	2015	Antonio Rueda et al.	Nucleic Acids Research https://www.ncbi.nlm.nih.gov/pmc/articles/PMC4489306/pdf/gkv555.pdf	10.1093/nar/gkv555 [94]
sRNAnalyzer	Web	MiRNA profiling strategies for isomiRs. Detection of potential SNPs in miRNAs. Uses Cutadapt, Printseq, FastX for preprocessing and Bowtie for mapping; MirBase and MirGen DB. Use of LPM (local probability mapping) strategy to increase mapping specificity. Extensive rRNA and tRNA filtering steps to enhance the accuracy of exogenous RNA mapping. Exogenous RNA mapping process using sRNAnalyzer.	2017	Xiaogang Wu et al.	Nucleic Acids Research Open Access https://www.ncbi.nlm.nih.gov/pubmed/29069500	10.1093/nar/gkx999 [95]
mirPRo	Linux	Quantify known miRNAs. Predict novel miRNAs and miRNA family expression quantification. IsomiRs identification and categorization. Arm switching detection. Using Novoalign, HTSeq, and pre-miRNAs from miRBase. Using DESeq to perform differential expression profile analysis for known and novel mature miRNAs.	2015	Jieming Shi et al.	Scientific Reports Open Access https://www.ncbi.nlm.nih.gov/pmc/articles/PMC4592965/	10.1038/srep14617 [96]
miRge	Standalone	MirGeneDB miRNAs were used to assemble positive clusters (known miRNAs and tRNA, snoRNA, rRNA or mRNA were used to assemble negative clusters (known non-miRNAs). Identification of isomiRs. Novel miRNA detection. Preprocessing with Cutadapt and mapping (Bowtie). Using miRbase and miRGen DB. Detection of A-to-I editing.	2015	Alexander S. Baras et al.	Plos one Open Access https://journals.plos.org/plosone/article?id=10.1371/journal.pone.0143066	10.1371/journal.pone.0143066 [97]
miRDis	Web	Systematic annotation of known miRNAs and other noncoding RNAs based on read mapped regions. Prediction of novel miRNAs and noncoding RNAs through assigning ambiguous reads to unique genome region with well-predicted RNA structure. Detection of candidate exogenous miRNAs transported from dietary species. Using FASTQC, Cutadapt, Bowtie, and EdgeR.	2017	Hanyuan Zhang et al.	Briefings in Bioinformatics Open Access https://www.ncbi.nlm.nih.gov/pubmed/28073746	10.1093/bib/bbw140 [98]
miRMOD	Windows	Identifies and analyzes miRNA 3′ and 5′ modifications (non-templated additions as well as trimming). Using Bowtie with reference genome/pre-miRNA. Provides useful statistics about various types of miRNA modifications along with its frequencies. Target alteration analysis.	2015	Abhinav Kaushik et al.	PeerJ Open Access https://www.ncbi.nlm.nih.gov/pubmed/26623179	10.7717/peerj.1332 [99]
mirTools 2.0	Web	Detection of various types of ncRNAs (miRNA, tRNA, snRNA, snoRNA, rRNA, and piRNA). Identify miRNA-targeted genes. Performs functional annotation or miRNA targets (GO, KEGG, PPI). Detect differentially expressed ncRNAs with RPM and the Bayesian method. Detect novel miRNAs using miRDeep.	2013	Jinyu Wu et al.	RNA Biology https://www.ncbi.nlm.nih.gov/pmc/articles/PMC3849156/	10.4161/rna.25193 [100]
Chimira	Web	Sequences are automatically cleaned, trimmed, size-selected, and mapped directly to miRNA hairpin sequences. Identifies epi-transcriptomic modifications in the input sequences. Alignment with BLAST to miRbase and differential expression of miRNAs using DESeq2. Modification profiles of 3′ and 5′ and internal modifications, uridylation, adenylation, and internal modifications or variations of the miRNAs.	2015	Dimitrios M. Vitsios et al.	Bioinformatics https://www.ncbi.nlm.nih.gov/pmc/articles/PMC4595902/	10.1093/bioinformatics/btv380 [101]
miRTOP	Standalone, Linux	Generation of a new file format mirGFF3. Compatible with the commonly used tools output files (sRNAbench, Prost!, isomiR-SEA, and OptimiR, e.g.). Converts mirGFF3 file into several different types such as count matrix, FASTA, and VCF formats. Open source and community-based project. API for the standardization of miRNA and isomiR annotation, enabling data sharing, reporting.	2019	Thomas Desvignes et al.	Bioinformatics Open Access https://academic.oup.com/bioinformatics/article/36/3/698/5556118	10.1093/bioinformatics/btz675 [102]
Prost!	Standalone, Linux	Aligns reads to a user-defined genomic dataset. Groups reads based on their potential genomic origins, seed sequence and annotation. Reports frequencies of individual sequence variations with respect to reference genome and the most expressed sequence. Uses Cutadapt and FASTX-toolkit for preprocessing, BBMap suite for alignment, miRBase for annotation, and DESeq2 for differential expression.	2019	Thomas Desvignes et al.	Scientific Reports Open Access https://www.nature.com/articles/s41598-019-40361-8	10.1038/s41598-019-40361-8 [103]
OptimiR	Standalone, Linux	Incorporates biological knowledge on miRNA editing and genome-wide genotype data Novel miRNAs and highlighting the allelic imbalance expression of polymiRs in heterozygous carriers. Uses Cutadapt for preprocessing, Bowtie2 for alignment, and miRBase for annotation. Uses a scoring algorithm to identify the most plausible alignments. Produces a comparison analysis of genotype data provided by the user and the genotype data that could be inferred from the sequenced reads aligned to polymiRs.	2019	Florian Thibord et al.	RNA Open Access https://rnajournal.cshlp.org/content/early/2019/02/28/rna.069708.118	10.1261/rna. 069708.118. [104]
isomiRs	R library	Uses as input file the count matrix. Includes various functions for characterization of miRNAs and isomiRs, clustering, differential expression, and visualizations. Designed to analyze the output of SeqBuster tool or any other tool after converting to the desire format [105].	2020	Pantano L., Escaramis G.	http://bioconductor.org/packages/release/bioc/html/isomiRs.html	10.18129/B9.bioc.isomiRs
